# Stacked hybridization to enhance the performance of artificial neural networks (ANN) for prediction of water quality index in the Bagh river basin, India

**DOI:** 10.1016/j.heliyon.2024.e31085

**Published:** 2024-05-11

**Authors:** Nand Lal Kushwaha, Nanabhau S. Kudnar, Dinesh Kumar Vishwakarma, A. Subeesh, Malkhan Singh Jatav, Venkatesh Gaddikeri, Ashraf A. Ahmed, Ismail Abdelaty

**Affiliations:** a Department of Soil and Water Engineering, Punjab Agricultural University Ludhiana, Punjab, 141004, India; b Division of Agricultural Engineering, ICAR-Indian Agricultural Research Institute, New Delhi, 110012, India; c Department of Geography, C. J. Patel College Tirora, Gondia, Maharashtra, 441911, India; d Department of Irrigation and Drainage Engineering, G.B. Pant University of Agriculture and Technology, Pantnagar, Uttarakhand, 263145, India; e ICAR- Central Institute of Agricultural Engineering, Bhopal, Madhya Pradesh, 462038, India; f National Institute of Hydrology, North Western Regional Centre, Jodhpur, Rajasthan, 342003, India; g Department of Civil and Environmental Engineering, Brunel University London, Kingston Lane, Uxbridge UB38PH, UK; h Water and Water Structures Engineering Department, Faculty of Engineering, Zagazig University, Zagazig, 44519, Egypt

**Keywords:** Groundwater, Water quality assessment, SVM, Water resources management, Machine learning

## Abstract

Water quality assessment is paramount for environmental monitoring and resource management, particularly in regions experiencing rapid urbanization and industrialization. This study introduces Artificial Neural Networks (ANN) and its hybrid machine learning models, namely ANN-RF (Random Forest), ANN-SVM (Support Vector Machine), ANN-RSS (Random Subspace), ANN-M5P (M5 Pruned), and ANN-AR (Additive Regression) for water quality assessment in the rapidly urbanizing and industrializing Bagh River Basin, India. The Relief algorithm was employed to select the most influential water quality input parameters, including Nitrate (NO_3_^−^), Magnesium (Mg^2+^), Sulphate (SO_4_^2−^), Calcium (Ca^2+^), and Potassium (K^+^). The comparative analysis of developed ANN and its hybrid models was carried out using statistical indicators (i.e., Nash-Sutcliffe Efficiency (NSE), Pearson Correlation Coefficient (PCC), Coefficient of Determination (R^2^), Mean Absolute Error (MAE), Root Mean Square Error (RMSE), Relative Root Square Error (RRSE), Relative Absolute Error (RAE), and Mean Bias Error (MBE)) and graphical representations (i.e., Taylor diagram). Results indicate that the integration of support vector machine (SVM) with ANN significantly improves performance, yielding impressive statistical indicators: NSE (0.879), R^2^ (0.904), MAE (22.349), and MBE (12.548). The methodology outlined in this study can serve as a template for enhancing the predictive capabilities of ANN models in various other environmental and ecological applications, contributing to sustainable development and safeguarding natural resources.

## Highlights

• A comparative study of ANN and its hybrid models (i.e., ANN-RF, ANN-SVM, ANN-RSS, ANN-AR, and ANN-M5P) was conducted for water quality assessment.
• Integrating SVM with ANN significantly improved the model performance.
• The new model can serve as a template for various environmental applications.
• This advancement holds promising decisions in water resource management.

## Graphical abstract

**Figure:**
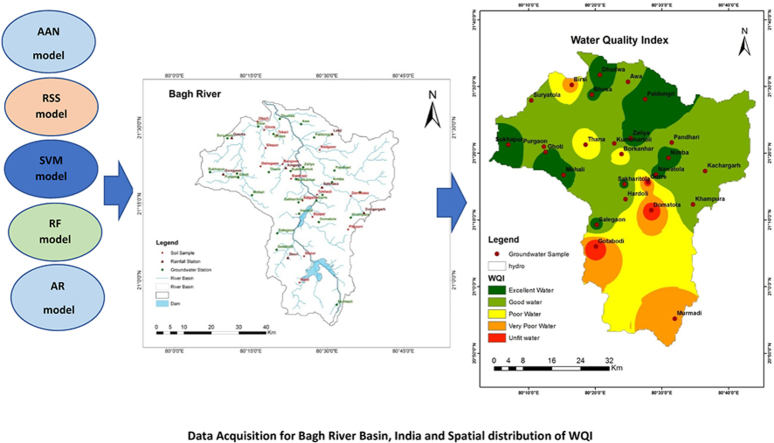

## 1 Introduction

Assessing and forecasting water quality holds significant importance in the realm of integrated water resource management. This domain recognizes groundwater as vital for human well-being and future progress [1]. The fundamental problem of managing water resources in stressful areas, particularly in developing nations [2,3]. Due to the release of contamination and its impact on the value of water properties globally, river basin water quality is an issue. The key to implementing methods for managing water resources in river basins and addressing the issue of river water pollution is to reduce river basin pollution by identifying the drivers and water quality metrics [4,5]. Since the industrial revolution, one of humanity's significant pertinent trials is the river water quality, which has been at high risk and deteriorating [6]. Predictive models are useful for evaluating the influence of hydrological and anthropogenic water stress on water value variables [7]. The lack of a shared blueprint for water quality data is a problem for most hydrological flux concentration databases, which produce relatively high time resolution [8]**.** In arid and semi-arid areas, water supplies are scarce while industry demands, drinking water, and agriculture are rising, particularly in areas experiencing drought [9,10].

Machine Learning (ML) models are effective methods for minimizing source quantification mistakes that cannot be avoided [11]. Additionally, the poorly understood biogeochemical and physical processes that drive the transport and transformation of pollutants are subject to fewer parameterization limits in the ML models. Machine learning is created to identify nonlinear behavior [12]. Artificial intelligence (AI) approaches are used more often in various fields. It is employed in hydrological forecasting and produces highly accurate river flow predictions [13]. Artificial intelligence is a good alternative and complements conventional methods for investigation and prediction. Using physical characteristics in groundwater resources irrigation water quality indexes (IWQI) is expensive and time-consuming for farmers, especially in developing nations [14]. Machine learning models are highly effective in reducing source quantification errors that cannot be eliminated by any other means [15].

To measure and assess the overall water quality index (WQI), Horton [16] suggested combining various factors into a single number. To estimate the suitability of groundwater for irrigation reasons using 13 physicochemical characteristics, Wagh et al. [17] utilized the artificial neural networks (ANN) model; the study revealed that ML models are quite accurate in predicting and examining water quality. Another study [18] in southeastern Nigeria leverages machine learning to enhance water quality analysis, a relatively unexplored area in the country. Employing integrated algorithms, the research accurately models groundwater quality, revealing 80 % of the resources as potable. Cluster analyses pinpoint contamination sources and spatial variations. Notably, both multiple linear regression and neural networks yield precise water quality predictions, underscoring their potential for advancing sustainable water management practices. Using k-means clustering in the major European rivers, Massei et al. [19] evaluated the impact of pesticides and biocides in river water on hazardous risk. To enhance the performances of individual models for the salinity and chlorophyll in beach water, particularly for multi-step ahead modeling, Shamshirband et al. [20] used multiple wavelets-ANNs models. Another study by Di et al. [21] developed classification ML models for IWQ prediction in the Yangtze River. Similarly, Ahmed et al. [22] provided a thorough review of different machine learning models used for water quality.

Water quality research has made significant progress in recent times, the use of various modeling approaches that have been applied to tackle different aspects of the issue. Castrillo and García (2020) utilized random forest (RF) and linear models to tackle high-nutrient levels in the river Thames. Meanwhile, Bui et al. [23] delved into WQI forecasting, exploring a combination of 4 conventional methods and 12 hybrid AI strategies. Their study showed that hybrid AI models outperformed conventional ones regarding predictive accuracy. Nafi et al. [24] introduced RF and random tree (RT) methods for classifying river water quality, considering parameters like thermal conductivity, temperature, total and fecal coliform concentrations, demand for biological oxygen, and nitrate. Agbasi and Egbueri [25] investigated water pollution in Umunya, Nigeria, using various indices like Human Health Risk (HHRISK), Modified heavy metal index (MHMI), Synthetic pollution index (SPI), and Entropy-weighted water quality index (EWQI),. Results show that 60 % of samples are safe for consumption, but 40 % pose risks, especially to children. Carcinogenic risks are high, and ingestion poses a greater risk than dermal contact. Artificial neural networks and multiple linear regression models provided precise predictions of water quality indices, while hierarchical dendrograms effectively categorized the water samples into different spatiotemporal water quality clusters. Jahin et al. [26] opted for multivariate analysis to study the IWQI for surface water in Egypt. Elbeltagi et al. [27] took a different approach by evaluating WQI at the Akot basin. They employed Support Vector Machine (SVM), random subspace (RSS), and additive regression (AR). Notably, the AR model was recommended due to its simplicity in terms of input parameters while maintaining reliability and accurate prediction.

In another study, Kouadri et al. [28] used a machine learning model to predict the water quality index (WQI) in Illizi, Southeast Algeria, particularly focusing on irregular data. They identified total dissolved solids (TDS) and total hardness (TH) as the main factors influencing WQI, with the mean absolute error (MAE) model proving to be the most accurate among the methods considered. Valentini et al. [29] developed a new WQI equation for Mirim Lagoon based on extensive data collected over three years at seven locations, with parameters including pH, dissolved oxygen, conductivity, turbidity, fecal coliform, and temperature. The study [30] in Pratapgarh, Southern Rajasthan, employs an artificial neural network (ANN) to predict groundwater sodium hazards for irrigation. Using MATLAB and ten years of data, the optimized ANN model effectively forecasts water quality indicators like sodium adsorption ratio (SAR), percent sodium (%Na) residual, Kelly's ratio (KR), and residual sodium carbonate (RSC). Finally, Shukla et al. [31] conducted a comparative analysis, evaluating a feed-forward artificial neural network (ANN) model against other algorithms. Their findings suggested that a more complex architecture involving the integration of the ANN algorithm with wavelets or an adaptive neuro-fuzzy reasoning system yielded superior results, particularly in accurately predicting stream flow in an Indian river.

Previous works indicated limited research focusing on developing hybrid machine learning models specifically tailored for predicting water quality, especially within the context of Indian conditions. In response to this gap, the present study delves into assessing the performance of various models, including Artificial Neural Networks (ANN) and its hybrid combinations, namely ANN-RF (Random Forest), ANN-SVM (Support Vector Machine), ANN-RSS (Random Subspace), ANN-M5P (M5 Pruned), and ANN-AR (Additive Regression). These models were applied to evaluate the Water Quality Index (WQI) in the Bagh River Basin, India. The primary objective of this study was not only to assess the performance of the ANN algorithms but also to enhance their predictive capabilities through hybridization with other machine learning algorithms. Bydoing so, we aimed to identify the most effective and suitable AI-based model for WQI prediction within the specific environmental conditions of the Bagh River Basin. It's crucial to note that the volume and organization of available data play a pivotal role in determining the effectiveness of various machine learning algorithms. Therefore, the selected algorithm ANN and its hybrids were chosen based on their proven track record of delivering robust performance and their aptitude for capturing dynamic, nonlinear relationships within datasets.

## 2 Methodology

### 2.1 Study area and available datasets

**Fig. 1:**
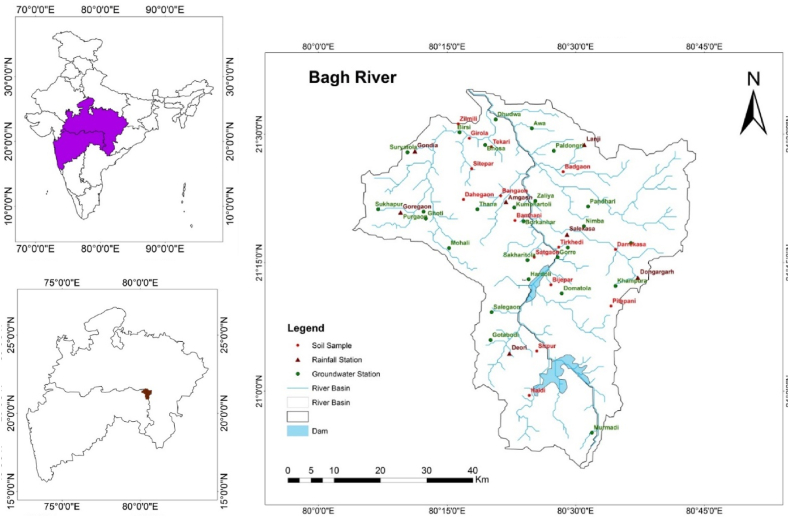
Case study river basin showing the location of water sample collected and river basin drainage networks.

The Bagh River is a significant tributary of the Wainganga River [32]. The river basin lies between latitude 20° 45′ 0″ N to 21° 45′ 0″ N latitude and longitude 80^0^ 00′ 0″ E to 80° 45′ 0″ E (Fig. 1). This river's axial and longitudinal extensions result in a total coverage area of 2876.9 Km2. This 130 km long river begins in the Cheezgad hills of the Sahyadri mountain range. Given the topography of this river, BRB is situated between the Wainganga River valley to the north, the Gaikhori hills to the west, the valleys to the east, and the Chichgad hills to the south. This river bed has an average elevation between 208 and 728 m. Two rivers, the Ghisari and Dev Rivers, on its right bank and the Pangoli river on its left, join this river. At Birsola in the Gondia District, the Bagh River merges with the Wainganga River.

Because metamorphic and igneous rocks cover the whole river basin, this research region is unlike any other in Maharashtra. The Pre-Cambrian Archaean Dharwars crystalline rocks make up a large portion, the Amgaon Group, which is limited to the northeast and northwest corners of the area surrounding Amgaon and Bahela, is the representative formation of the Archeans [33]. It is made up of Augen gneisses, amphibolites, and migmatites. The Sakoli Group and Dongargarh Group of rocks, which together comprise the main stratigraphic block, is representative of the Lower Precambrian Dharwars, which come after the Amgaon group. The Sakoli Group is limited to the northern and western regions of Nagjhira and is made up of quartzites, schists, phyllites, metavolcanics, and BIF. Rhyolites, Andesites, and basic volcanics are found in the vicinity of Salekasa, Wadegaon, Murdoli, Deori, and Chinchgarh. These rocks correspond to the Dongargarh Group's Bijli, Pitepani, and Sitagota formations [33,34].

Groundwater samples were taken from 26 wells in the Bagh River basin during the pre-monsoon season, and analyses were done for the different perimeters. Composite sampling is carried out when the liquid matrix is expected to be heterogeneous and varies from time to time or depth or at many sampling locations. This type of sampling provides a representative sampling for this type of matrix and is carried out by combining portions of multiple grab samples collected at regular intervals. If the flow is expected to be constant, then volume-based sampling can be carried out. If the flow varies, like sewerage line, then sampling can be done by flow-based composite, i.e., collecting sample that is proportional to the discharge. Time composite sampling represents a 24- hour period, with interval being 1–3 h. Use composite samples only for parameters that will remain unchanged under the sampling conditions, preservation and storage. The factors listed here consist of pH, Sodium (Na^+^), Sulphate (SO_4_^2−^), Bicarbonate (HCO3^-^), Total dissolved solids (TDS), Total Hardness (TH), Magnesium (Mg^2+^), Chloride (Cl^−^), Calcium (Ca^2+^), Nitrate (NO_3_^−^), and Fluoride (F^−^). Collection, preservation, transportation, storage, and weighted arithmetic index method analysis of the sample.

### 2.2 Computation *of the water quality index (WQI)*

The evaluation of groundwater quality for irrigation purposes is based on the WQI, which is frequently used to evaluate water quality and its suitability for agricultural use [3,35]. The WQI is a comprehensive rating system that considers various water quality variables and condenses them into a single overall rating, representing the overall water quality. In this study, ten significant characteristics were considered to compute the WQI. The first phase necessitates giving unit weights to each physicochemical parameter using a "weighted arithmetic index" to normalize the parameters with different units and dimensions onto a comparable scale [36].

The proportional weights for each parameter were determined based on their unit weights. The quality rating was computed by comparing each parameter's observed concentration and norm concentration. The sub-index was then produced by multiplying the quality rating of each parameter by the appropriate relative weight. The WQI, which was the result of adding the sub-indices for each attribute, was then developed. More details about the assigned weights (Wi), relative weights (wi), and the WHO standard are provided in Table 1 [37]. The assigned weights (Wi), is calculated using equation (1) given below:

(1) $$W_{i}=\frac{w_{i}}{\sum_{i=1}^{n}w_{i}}$$

**Table 1:** Weight of parameters and their standard for WQI.

Chemical parameters	Standards (BIS 2003 [37];	Weight (w_i_)	Relative weight (W_i_)
Sulphate (SO_4_^2−^)	200	5	0.114
Nitrate (NO_3_^−^)	45	5	0.114
Fluoride (F^−^)	1	5	0.114
Chloride (Cl^−^)	250	5	0.114
Total dissolved solids (TDS)	500	5	0.114
Sodium (Na^+^)	50	5	0.114
pH	8.5	3	0.068
Calcium (Ca^2+^)	75	3	0.068
Magnesium (Mg^2+^)	30	3	0.068
Potassium (K^+^)	100	2	0.045
Total hardness (TH)	300	2	0.045
Bicarbonate (HCO_3_^−^)	200	1	0.023
		Ʃ wi = 44	Ʃ Wi = 1

Note: All concentrations in given mg/L excluding pH.

A quality rating scale (qi) is calculated for each parameter by using equation (2) given as:

(2) qi=(CiSi)×100

Additionally, a subindex of the ith parameter is estimated based on equation (3) given as:

(3) $${SI}_{i}=q_{i}\times W_{i}$$

Lastly, the water quality index is calculated using equation (4) given as:

(4) $$WQI=\sum {SI}_{i}$$

where $W_{i}=$ relative weight, $w_{i}=$ weight/parameter, n= number of parameters, $C_{i}=$ chemical concentration per water sample (mg/L), $S_{i}=$ quality standard for drinking water as per WHO (mg/L), ${SI}_{i}=$ subindex rating, $q_{i}=$ quality rating and $W_{i}=$ relative weight

### 2.3 Machine learning algorithms

#### 2.3.1 Artificial neural network (ANN)

**Fig. 2:**
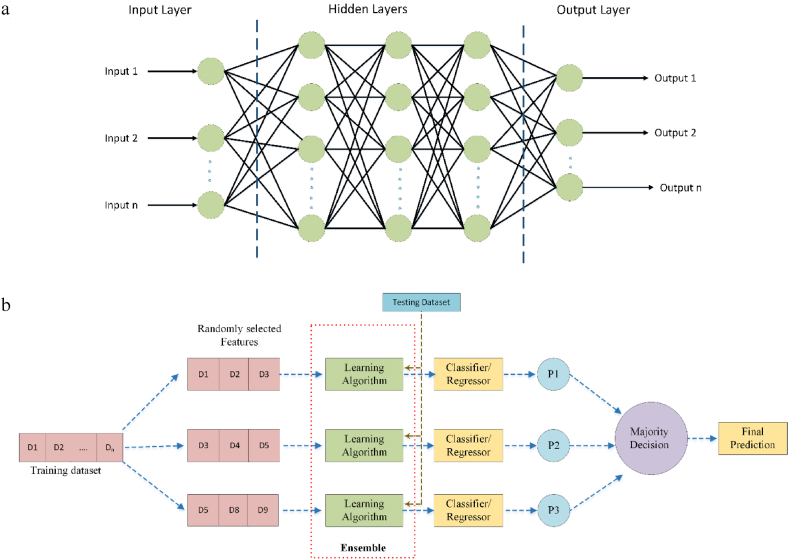
Schematic diagram of (a) ANN (b) Random subspace method.

Artificial Neural Network (ANN) is a computational modeling tool containing interconnected adaptive dispensation rudiments, capable of executing massive parallel computations for complex data processing and knowledge representations [[38], [39], [40]]. In the past few decades, research into ANNs has shown explosive growth, covering various applications in various areas. ANN models follow an exact planning, which the biological nervous system enthuses. Like the human brain, the ANN model comprises neurons arranged in a complex nonlinear form in a layered fashion, and the neurons in adjacent layers are interconnected by weighted links [41]. Each input is multiplied by its appropriate weights after being received by the input layer of the ANN in the form of text, numeric, or picture vectors. These weights often reflect how strongly the ANN's neurons are connected. The middle, hidden layer(s) performs mathematical computations to extract patterns from the input data. The hidden layer's meticulous computations enable the ANN to produce the desired result in the output layer. The architecture of ANN is shown in Fig. 2a. Ideally, ANNs are trained with large datasets to derive meaningful insights and patterns from the dataset [42].

#### 2.3.2 Random subspace (RSS)

The random subspace algorithm is a machine learning ensemble method that enhances diversity among ensemble learners by limiting the models to operate on various random subsets of the entire feature space [43,44]. The general layout of RSS is presented in Fig. 2b. The issue of very large dimensionality is elegantly solved with RS ensembles. Smaller subspaces make it easier to train the predictors and significantly increase the feature-to-instance ratio [45]. When there are few training items in proportion to the amount of data, RSS is extremely useful. Furthermore, random subspace offers stronger predictors when data contains many redundant features than the original feature space. The first phase entails predicting the initial space into subsets, and in the final stage, the result obtained is aggregated through voting or averaging [46].

#### 2.3.3 Support vector machine (SVM)

**Fig. 3:**
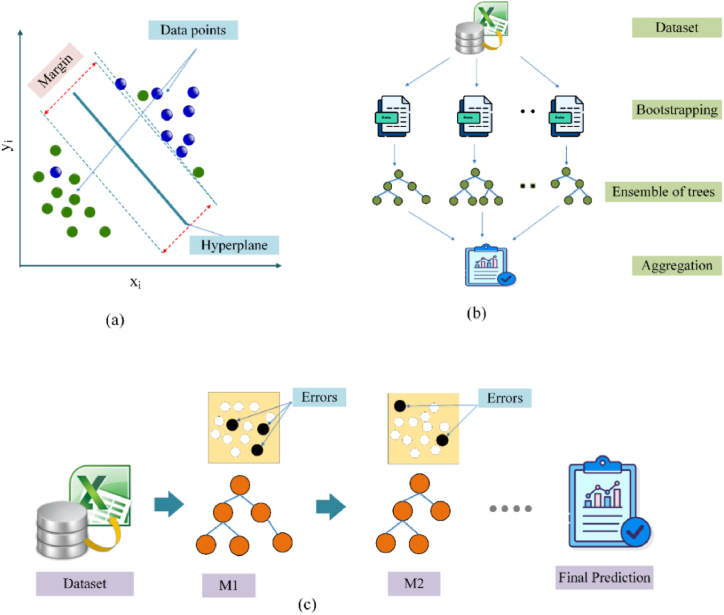
Schematic diagram of (a) SVM, (b) Random Forest, and (c) Additive Regression models.

Supervised learning is a popular classification method, and regression and outlier detection is the support vector machine. The classification job serves as the greatest lens to comprehend the SVM algorithm. In an N-dimensional space, the SVM classifier creates a hyperplane that divides the data points into different classes [[47], [48], [49]]. The margin is used to choose the hyperplane. In other words, the hyperplane with the largest margin between the classes is picked. Support vectors-data points closer to the hyperplane are used to determine these margins. SVM can be well utilized as a regression approach, maintaining all the key topographies that describe the algorithm (maximal margin). SVM is well suited for regression issues due to its sparse solution and stronger generalization ability (Fig. 3a). A new *ε*-insensitive region, known as *ε*-tube generated around the function, helps approximate the continuous-valued function and reduces the prediction error. Like SVM classifiers, the support vectors are the most important factors affecting how the tube is shaped in SVR. SVR also counts on the independence and identical distribution of the training and testing sets of the data [50].

#### 2.3.4 Random forest (RF)

The popular and adaptable supervised machine learning technique Random Forest is effective for classification and regression issues. The core idea behind RF is to grow and combine multiple decision trees to form a “forest.” All choice tree in a random forest is trained on a subset of data, and the contribution of individual trees gives stability to the algorithm and reduces the variance [51,52]. The algorithm creates individual trees from different input data samples; further, at each bulge, dissimilar samples of topographies are designated for excruciating. The trees run in similar deprived of any interaction, and finally, the prediction from individual trees is averaged to produce the final result for the random forest regressor prediction. RF replicas have remained proven to be robust forecasters for both small datasets and higher dimensional data [53]. RF exhibits better generalization and tends to outpace most additional methods in footings of their performance, deprived of overfitting. Compared to decision trees, RF is more robust to noise in the dataset, and hyperparameter tuning is relatively easy [54]. The general layout of RF is presented in Fig. 3b.

#### 2.3.5 Additive regression (AR)

The additive regression model performs stage-wise addition, and new learners are extra one at a period by freezing the existing learners. i.e., the previous learners are left unchanged. A collaborative of feeble regression prediction models, often decision trees, is produced by additive regression as a prediction model. The additive regression trees are very similar to the gradient boosting trees, wherein contributions of sequential weak learners are strengthened at each iteration. In every iteration, it fits a model to the residuals of the previous iteration. The model's residuals are used for training, which gives the incorrectly predicted data more weight. Additionally, each weak learner's contribution to the final prediction is based on a gradient optimization technique to lower the overall error of the strong learner.

The overfitting is prevented by reducing the learning rate parameter and providing a smoothing effect [55]. With vast and complex datasets, these additive regression stands out for their accurate prediction capabilities [56]. The architecture of AR is shown in Fig. 3c.

#### 2.3.6 M5 pruned (M5P)

The M5 tree algorithm, introduced by Quinlan [57] is a choice tree with linear regression at the leaf nodes, that can help predict incessant arithmetical qualities. The M5P algorithm is simple to apply and gives more comprehensible linear mathematical equations among the contribution and yield variables when likened to additional machine learning algorithms. The model efficiently predicts continuous values and can handle data with higher dimensionality. The computation of error at each node provides the basis for determining the excruciating standard for the M5P model tree. The error is analyzed based on the standard deviation of the standards at a particular node. The data in child nodes are purer and have a lower standard deviation than that of the parent node due to the splitting process. The model evaluates each alternative split, choosing the one that minimizes errors while maximizing error reduction [58]. This approach often creates a huge tree-like structure that could lead to overfitting. The overgrown trees are pruned to tackle this overfitting by relieving the sub-trees with linear regression functions [59].

#### 2.3.7 Selection of best input combination for model development

**Fig. 4:**
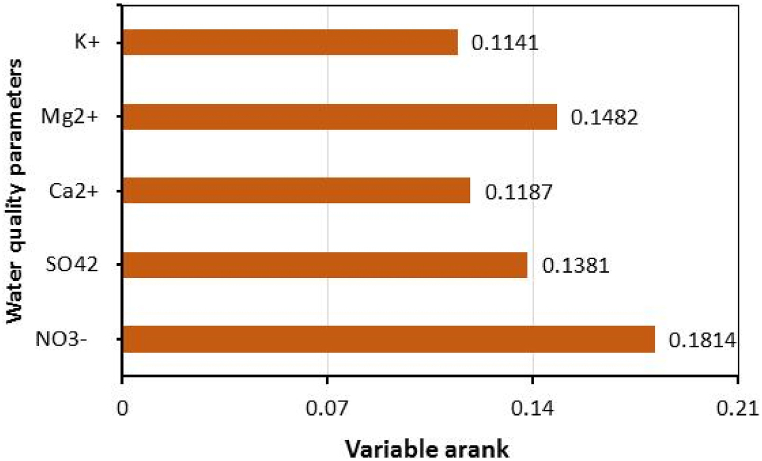
Weightage of selected variables for model development.

The best performance of the selected models depends on carefully selecting the water quality input parameters during the water quality modeling process. Numerous combinations of these parameters were utilized to find the ideal input combination. Then, using the Relief method, a certain combination was found to be the best [60]. The relief algorithm has emerged as a widely adopted technique for feature selection. Its primary objective is to assess the significance of individual features within a dataset by gauging their capacity to differentiate between distinct classes. The operational principle of this algorithm revolves around attributing weights to each feature, predicated on their effectiveness in distinguishing between neighboring instances within the feature space [61]. The algorithm's functionality can be summarized as follows: It assigns weight values to features based on their aptitude for discriminating among closely situated data points within the feature space. These weight values subsequently undergo a prioritization process, leading to the ranking of features based on their perceived importance. Features that attain higher ranks are deemed more pertinent in contributing to the differentiation of classes. Utilizing the relief algorithm confers multiple advantages, notably in scenarios where the novel dataset includes many structures. By electing to retain the most pertinent features according to the algorithm's ranking, it becomes possible to enhance the correctness and efficacy of machine learning models. This is predominantly beneficial in situations where the volume of features might otherwise introduce complexity and resource-intensive computations [3,62]. Among 12 independent input variables, i.e., pH, HCO_3_^−^, Cl^−^, NO_3_, TDS, TH, Ca^2+^, Mg^2+^, Na^+^, K^+^, SO_4_^2−^ and F^−^), the five most influencing variables were selected for model development. These include NO_3_^−^, SO_4_^2−^, Ca^2+^, Mg^2+^, and K+. Fig. 4 presents the ranks of the selected variables for predicting the WQI.

#### 2.3.8 Fusion of meta-heuristic algorithms through stacked generalization

Stacked hybridization, also known as stacked ensemble learning, is a machine learning technique that combines multiple diverse machine learning models to improve predictive performance [63]. This approach leverages the strengths of individual models and mitigates their weaknesses by training a meta-model, or a "stacked" model, on the predictions made by these base models. The stacked model learns how to weigh the predictions from each base model to make a final prediction, often resulting in improved accuracy, robustness, and generalization. Research findings indicate that using stacked hybrid algorithms can enhance the predictive capabilities of these algorithms [64,65]. Stacked hybridization allows you to take advantage of the diverse strengths of different models, potentially leading to improved predictive performance compared to using any single model in isolation. However, it's essential to perform careful model selection, tuning, and validation to ensure the success of a stacked ensemble. The steps involved in the stacked hybridization of an Artificial Neural Network (ANN) with another machine learning algorithm, such SVM, are outlined below.

**Step 1:** Begin by selecting two base models: base model 1, which is the ANN, and base model 2, which is the SVM.
**Step 2:** Split the training data into two sets: training the ANN and SVM (the first-level training data) and training the stacked model (the second-level training data).
**Step 3:** Train the ANN using the first-level training data while adjusting the neural network's architecture and parameters. Simultaneously, train the SVM using the first-level training data while optimizing the kernel and hyperparameters.
**Step 4:** Employ the trained ANN and SVM to make predictions on a validation or holdout dataset.
**Step 5:** Train a meta-model, such as logistic regression or a decision tree, utilizing the predictions generated by the ANN and SVM on the validation dataset. This meta-model is designed to learn how to effectively combine these predictions.
**Step 6:** For making predictions on new data, apply both the ANN and SVM to generate predictions. Then, employ the trained meta-model to combine these predictions, resulting in the final prediction.

### 2.4 Evaluation of the statistical performance of hybrid model developments

The evaluation of the performance of the computed Water Quality Index (WQI) and predicted WQI using hybrid models involved the utilization of commonly recognized statistical metrics. These metrics encompass the Nash-Sutcliffe efficiency (NSE), Pearson correlation coefficient (PCC), Coefficient of determination (R2), Mean absolute error (MAE), Root mean square error (RMSE), Relative root square error (RRSE), Relative absolute error (RAE), and Mean Bias Error (MBE). These metrics have been effectively employed to assess model performance in previous studies [[66], [67], [68], [69]]. The RMSE is employed to quantify the disparity between expected and observed values within a time series. RRSE, as the square root of relative squared error, minimizes errors in dimensions that match the predicted quantity. MAE describes the mean absolute deviation of anticipated time series values from observed values. RAE assesses the absolute error's magnitude relative to the measurement's size and displays the ratio of absolute error to the actual measurement. Nash-Sutcliffe efficiency is a widely used statistic for evaluating model performance, ranging from 1, indicating an ideal fit, to −1. A value of 0 implies accuracy equivalent to the mean value.

On the other hand, the coefficient of determination (R^2^) quantifies the linear relationship between dependent and independent variables. In the context of WQI modeling, models with higher R2 values (closer to 1), higher RRSE values, and lower values of MBE, RMSE, MAE, and RAE are considered superior. In equations (5), (6), (7), (8), (9), (10), (11), the ${WQI}_{C}$ and ${WQI}_{P}$ represent the computed/observed and predicted or simulated values for the ith dataset, while ${WQI}_{cavg}$ and ${WQI}_{pavg}$ denote the average or mean magnitude of observed and predicted or simulated values. N signifies the number of observations.

(5) $$MBE=\frac{1}{N}\sum_{i=1}^{N}\left({WQI}_{P}-\;{WQI}_{C}\right)$$

(6) $$RMSE=\sqrt{\frac{1}{N}\sum_{i=1}^{N}{\left({WQI}_{C}-\;{WQI}_{P}\right)}^{2}\;}$$

(7) $$RRSE=\sqrt{\frac{\sum_{i=1}^{N}{\left({WQI}_{C}-\;{WQI}_{P}\right)}^{2}}{\sum_{i=1}^{N}{\left({WQI}_{C}-{WQI}_{cavg}\right)}^{2}}\;}$$

(8) $$MAE=\frac{1}{N}\sum_{i=1}^{N}\left|{WQI}_{C}-\;{WQI}_{P}\right|$$

(9) $$RAE=\frac{\sum_{i=1}^{N}\left|{WQI}_{C}-\;{WQI}_{P}\right|}{\sum_{i=1}^{N}\left|{WQI}_{C}-{WQI}_{cavg}\right|}$$

(10) $$R^{2}=1-\frac{\sum_{i=1}^{N}{\left({WQI}_{C}-\;{WQI}_{P}\right)}^{2}}{\sum_{i=1}^{N}{\left({WQI}_{C}-{WQI}_{cavg}\right)}^{2}}$$

(11) $$NSE=1-\left[\frac{\sum_{i=1}^{N}{{WQI}_{C}-\;{WQI}_{P})}^{2}}{\sum_{i=1}^{N}{\left({WQI}_{C}-{WQI}_{cavg}\right)}^{2}}\right]$$

## 3 Results

### 3.1 Dominance analysis and relative importance of water quality parameters

The dominance analysis of water quality input parameters uses the Relief algorithm [60]. Fig. 4 presents the ranks of the selected variables (i.e., NO_3_, Mg^2+^, SO_4_^2−^, Ca^2+^, and K^+^) from 12 water quality parameters (i.e., pH, HCO_3_^−^, Cl^−^, NO_3_, TDS, TH, Ca^2+^, Mg^2+^, Na^+^, K^+^, SO_4_^2−^ and F^−^) for predicting the WQI. The detailed analysis of the chemical composition of water quality is summarized in Table 2. The values of pH ranged from 6.60 to 8.92 with an average of 7.73 ± 0.52; TDS varies from 241 to 2100 with an average of 678 ± 469.94 and 30.0 to 681.0 with an average of 246.54 ± 176.98 for TH. Among cations, their concentration ranged from 7.80 to 680.0 with an average of 293.65 ± 193.43 for Na^+^; 0.20 to 411.0 with an average of 57.58 ± 106.76 for K^+^; 1.20 to 241.0 (100.16 ± 74.46) for Ca^+^, and 1.22 to 161.24 with an average of 51.70 ± 47.82 for Mg^+^. However, their anion attentiveness alternated from 14.0 to 3014.80 with an average of 472.67 ± 615.08 for Cl^−^; 128.0 to 652.0 with a normal of 293.65 ± 123.01 for HCO_3_^−^ and 6.0 to 481.0 with an average of 75.07 ± 127.95 for SO4^2−^. In footings of anions, Chloride is the maximum predominant, shadowed by Bicarbonate and Chlorine. The weightage of selected water quality parameters for WQI prediction has been shown in Fig. 4.

**Table 2:** Statistical summary of water quality parameters.

Parameters	Mean	SD	Skewness	Kurtosis	Minimum	Maximum	Range	WHO (1997)		BIS (2003) (IS 10500)	
								Maximum desirable	Highest permissible	Maximum desirable	Highest permissible
pH	7.73	0.52	−0.12	0.60	6.60	8.92	2.32	7.0–8.5	6.5–9.2	6.5–8.5	8.5–9.2
TDS	678.00	469.94	1.98	4.39	241.00	2100.00	1859.00	500	1500	500	2000
TH	246.54	176.98	1.09	0.91	30.00	681.00	651.00	100	500	300	600
Ca^+2^	100.16	74.46	0.54	−0.82	1.20	241.00	239.80	75	200	75	200
Mg^+2^	51.70	47.82	1.05	0.28	1.22	161.24	160.02	30	150	30	100
Na^+^	293.65	193.43	0.43	−0.72	7.80	680.00	672.20	50	200	–	–
K^+^	57.58	106.76	3.09	8.85	0.20	411.00	410.80	100	200	–	–
HCo3^-^	293.65	123.01	1.72	4.51	128.00	652.00	524.00	200	600	200	600
Cl^−^	472.67	615.08	3.02	11.65	14.00	3014.80	3000.80	250	600	250	1000
No_3_^−^	61.38	197.66	4.13	18.28	0.11	957.80	957.69	–	50	45	100
SO4^−2^	75.07	127.95	2.70	6.96	6.00	481.00	475.00	200	600	200	400
F^−^	0.97	0.70	0.04	−1.36	0.06	2.10	2.04	0.6–1.5	1.5	1.0	1.5

Note: All concentrations in mg/L, excluding pH.

### 3.2 Prediction of water quality index (WQI)

The primary objective of this study is to create innovative hybrid machine learning algorithms/models and assess their predictive capabilities for the Water Quality Index (WQI) in the Bagh River Basin (BRB). This section presents the outcomes of modeling WQI using data-driven hybrid machine-learning algorithms. We evaluated the performance of the Artificial Neural Network (ANN) and its hybridization with five other machine learning algorithms: ANN-RF, ANN-SVM, ANN-RSS, ANN-AR, and ANN-M5P, for WQI prediction.

#### 3.2.1 Development of models and their training

We investigated the enhancement of artificial neural networks (ANN) through stacked hybridization with other machine learning algorithms to improve water quality prediction. Water quality parameters, notably K^+^, Ca^2+^, SO_4_, Mg^2+^, and NO_3_^−^, were identified as the most influential input factors for WQI prediction. To assess the performance of the hybridized models relative to the conventional ANN, we employed eight statistical indicators to evaluate each model's effectiveness. The results obtained during the training phase are summarized in Table 3.

**Table 3:** Statistical indices of the proposed hybrid models during the training.

Statistical indices	ANN	ANN-RF	ANN-SVM	ANN-RSS	ANN-AR	ANN-M5P
PCC	0.996	0.984	0.956	0.996	0.977	0.996
R^2^	0.991	0.968	0.913	0.992	0.954	0.992
MAE	9.435	15.777	29.431	20.889	18.558	13.029
MBE	3.289	−0.850	0.000	−15.223	−4.556	−11.608
RMSE	11.695	20.229	40.961	29.332	25.583	17.351
RAE (%)	10.185	17.032	31.772	22.551	20.034	14.065
RRSR (%)	10.302	17.821	36.083	25.839	22.536	15.285
NSE	0.989	0.968	0.870	0.933	0.949	0.977

Table 3 illustrates that the ANN model did remarkably well to predict training results during the prediction phase, as the Pearson's correlation coefficient (PCC) for ANN was 0.996. The performance indicators showed the smallest values with an MAE = 9.435, MBE = 3.289, RMSE = 11.695, RAE (%) = 10.185 and RRSR (%) = 10.302, and the highest value of NSE for ANN was 0.989. It was trailed straight by the ANN-M5P model which had a Pearson's correlation coefficient of PCC = 0.996, smallest values of MAE = 13.029, MBE = −11.608, RMSE = 17.351, RAE (%) = 14.065 and RRSR (%) = 15.285. The highest value of NSE for ANN-M5P was 0.977, while the nethermost accomplishment model in the exercise stage was the ANN-SVM model with Pearson's correlation coefficient (PCC) = 0.956, and smallest values of MAE = 29.431, MBE = 0.000, RMSE = 40.961, RAE (%) = 31.772 and RRSR (%) = 36.083, and the highest value of NSE for ANN-SVM was 0.870. Grounded on the numerical presentation indicators acquired throughout the exercise phase of all seven models, it was obvious that they performed well.

This further demonstrated that in the training data sets, the ANN model outperformed the ANN-M5P, ANN-RF, ANN-AR, ANN-RSS, and ANN-SVM models in predicting WQI. During the training phase, the ANN-SVM model performs noticeably poorer at predicting the WQI. The top four models, ANN, ANN-M5P, ANN-RF, and ANN-AR, were chosen to forecast WQI because of their excellent precision and accuracy.

**Fig. 5:**
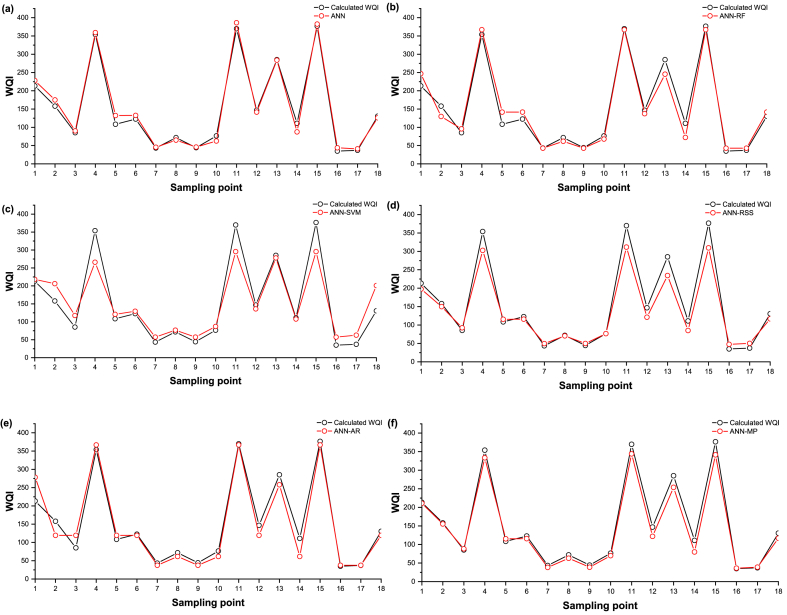
Line diagram of computed and predicted WQI for training data sets for (a) ANN stand-alone, (b) ANN-RF, (c) ANN-SVM (d) ANN-RSS, (e) ANN-AR, and (f) ANN-M5P.

**Fig. 6:**
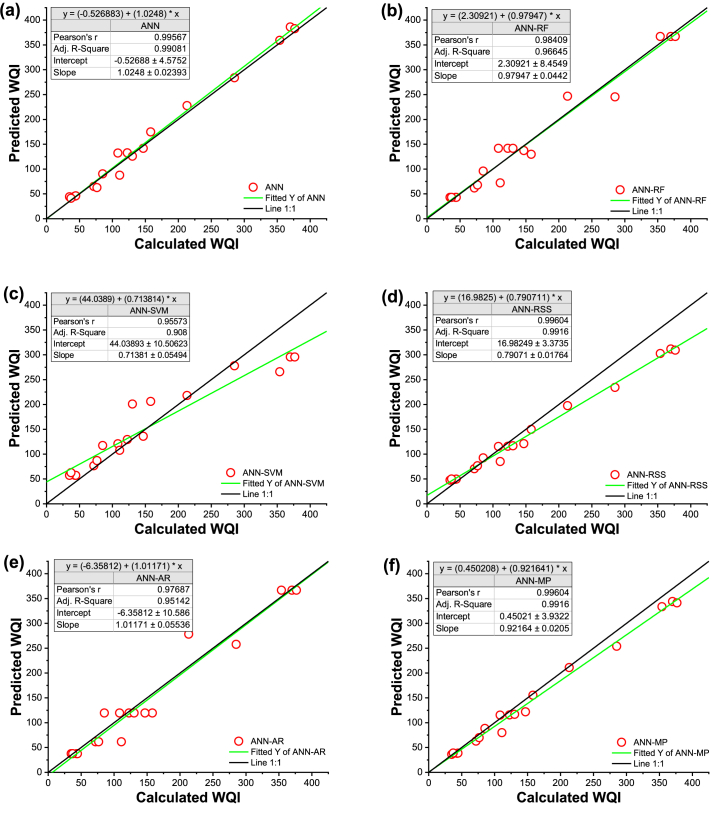
Scatter plot of computed and predicted WQI for training data sets (a) ANN stand-alone, (b) ANN-RF, (c) ANN-SVM (d) ANN-RSS, (e) ANN-AR, and (f) ANN-M5P.

In the training phase, the contrast between observed and predicted WQIs was presented using time series and scatter plots to illustrate the comparison between observed and predicted WQI based on the selected models (Fig. 5, Fig. 6). In Fig. 5, the simulations by ML models (continuous red line with circle symbol) are compared with the calculated WQI (continuous black line with circle symbol). The period sequence in this study was constructed from the time series generated by all sampling sites based on the training data set. Statistical parameters (i.e., MBE), line diagram (Fig. 5), and scatter plot (Fig. 6) show that the ANN was slightly over-predictive than the others.

When all the model's values are evenly spaced along or on either side of the 1:1 line, suggesting errors in the data, that model is shown to be accurate. In contrast to the values predicted by the ANN-RF, ANN-SVM, ANN-RSS, ANN-AR, and ANN-M5P models, which are all dispersed under the 1:1 line, the values predicted by the ANN model (R^2^ = 0.991) are more equally distributed over the 1:1 line. ANN-SVM and ANN-RSS are shown to be more under-predictive than others.

Our analysis of the performance values of the indicators showed that the eight models, on the whole, perform at an acceptable level. Yaseen et al. [13] and Markuna et al. [70] found that the RMSE is one of the most significant quantitative indicators of model performance during any analysis of data-mining models and time series data forecasting since it is one of the most predictive indicators.

#### 3.2.2 Validation of applied ML algorithms

Table 4 provides a summary of the results obtained during the validation phase. Among the models tested, the ANN model displayed the highest correlation and the lowest error during the training phase. However, its performance with the test datasets was suboptimal. On the other hand, the proposed hybrid ANN-SVM model exhibited the lowest error indicators and the highest Pearson's correlation coefficient (PCC = 0.951) during the validation phase. Notably, it achieved high values for NSE (0.879), PCC (0.951), and R^2^ while demonstrating low values for MAE (22.349), MBE (12.548), RMSE (27.974), RAE (30.039 %), and RRSR (34.227 %). These results indicate that the ANN-SVM model effectively recognized the WQI pattern and provided accurate predictions.

**Table 4:** Statistical indices of the proposed model in the testing datasets.

Statistical indices	ANN	ANN-RF	ANN-SVM	ANN-RSS	ANN-AR	ANN-M5P
PCC	0.923	0.880	0.951	0.927	0.910	0.927
R^2^	0.852	0.774	0.904	0.859	0.828	0.859
MAE	18.362	33.855	22.349	24.552	34.247	22.261
MBE	−7.944	−29.733	12.548	−20.809	−34.247	−20.579
RMSE	31.923	49.224	27.974	40.804	48.405	37.499
RAE (%)	24.680	45.502	30.039	32.999	46.029	29.920
RRSR (%)	39.059	60.228	34.227	49.925	59.226	45.881
NSE	0.842	0.625	0.879	0.742	0.637	0.782

The ANN model closely follows the top-performing analytical model, ANN-SVM. The ANN model achieved high values for NSE (0.842), PCC (0.923), and R^2^ (0.852) and displayed low values for MAE (18.362), MBE (−7.944), RMSE (31.923), RAE (24.680 %), and RRSR (39.059 %). Additionally, the ANN-M5P model exhibited strong performance with high NSE (0.782), PCC (0.927), R^2^ (0.859), and low MAE (22.261), MBE (−20.579), RMSE (37.499), RAE (29.920 %), and RRSR (45.881 %). In contrast, the ANN-RF model showed less favorable test results with PCC = 0.880, R^2^ = 0.774, MAE = 33.855, MBE = −29.733, RMSE = 49.224, RAE (%) = 45.502, and RRSR (%) = 60.228, along with an NSE of 0.625. These results clearly indicate that the ANN-SVM model outperformed the ANN, ANN-M5P, ANN-RSS, ANN-AR, and ANN-RF models in predicting WQI for the test datasets. The noticeably poorer performance of the ANN-RF model during the testing phase suggests that the inconsistent quality of the test dataset may have contributed to its subpar results.

**Fig. 7:**
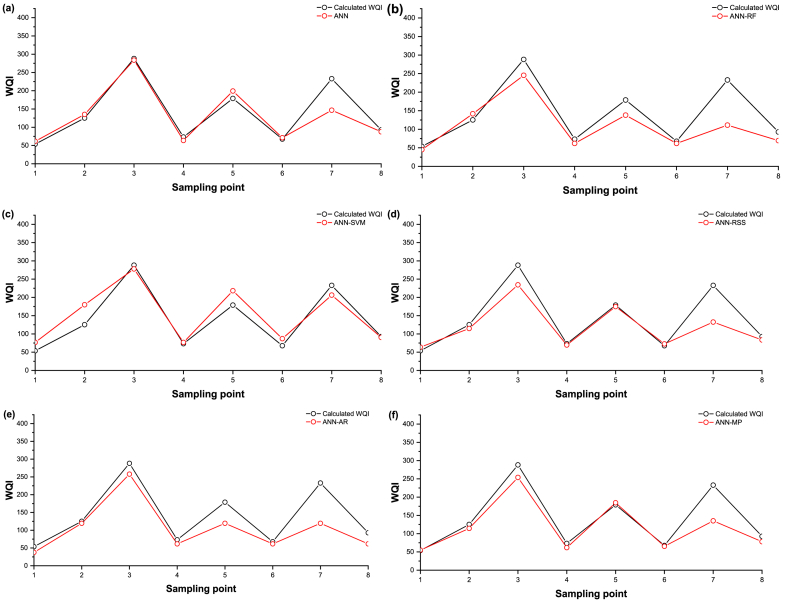
Line diagram of computed and predicted WQI for testing data sets for (a) ANN stand-alone, (b) ANN-RF, (c) ANN-SVM (d) ANN-RSS, (e) ANN-AR, and (f) ANN-M5P.

**Fig. 8:**
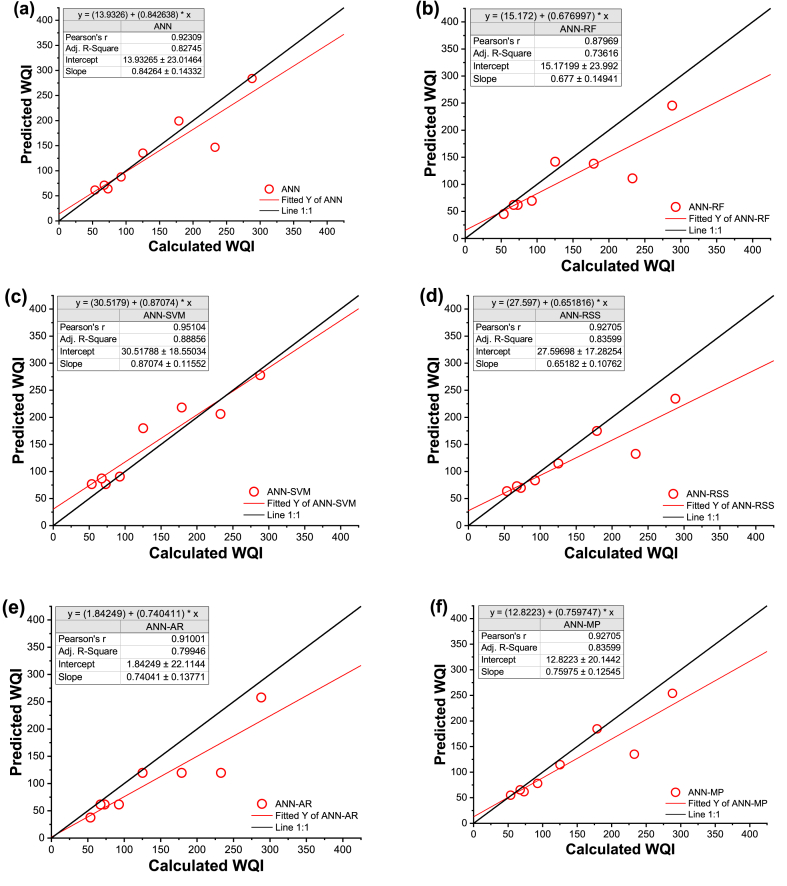
Scatter plot of computed and predicted WQI for testing data sets for (a) ANN stand-alone, (b) ANN-RF, (c) ANN-SVM (d) ANN-RSS, (e) ANN-AR, and (f) ANN-M5P.

To visualize the disparities between observed and predicted WQI based on the selected models, we compared them using time series and scatter plots during the validation phase (Fig. 7, Fig. 8). In Fig. 7, the simulations by ML models (represented by the continuous red line with circle symbols) were contrasted with the computed WQI (shown as the continuous black line with circle symbols). The time series used in this study was constructed from data generated by all sampling sites based on the testing dataset.

Statistical parameters, such as MBE, along with the line diagram (Fig. 7) and scatter plot (Fig. 8), indicated that the ANN-SVM model exhibited a slightly higher level of over-prediction than the other models. An accurate model typically exhibits an even distribution of values on or around the 1:1 line, signifying a balanced representation of errors. However, the values predicted by the ANN-SVM model (R^2^ = 0.904) were notably more evenly distributed along the 1:1 line compared to the predictions of the ANN, ANN-RF, ANN-RSS, ANN-AR, and ANN-M5P models, which all showed a dispersion below the 1:1 line, as evident in Fig. 8. Except for ANN-SVM model, all other models tended to under-predict the observed values.

**Fig. 9:**
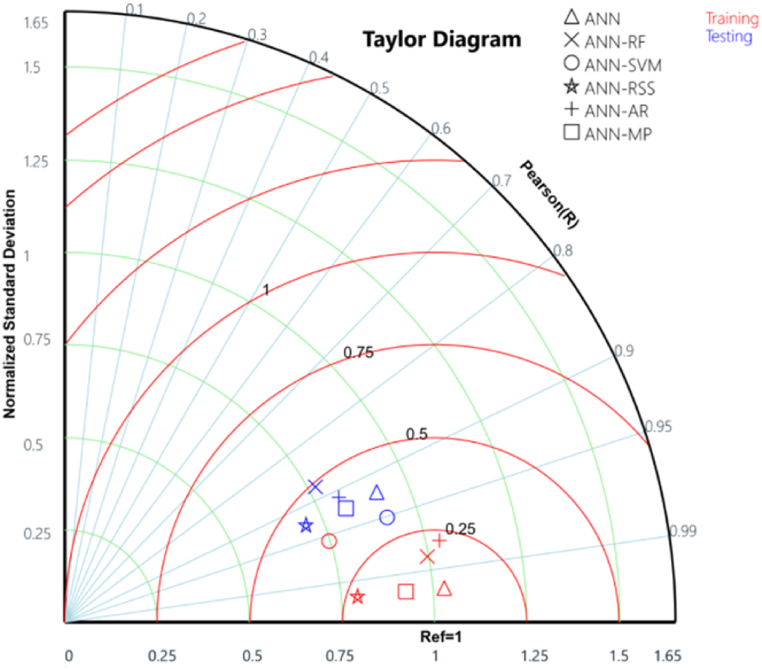
Taylor diagram showing comparative performance of developed hybrid models.

In addition, a Taylor diagram was employed to assess the model's performance, as introduced by Ref. [71]. Fig. 9 illustrates that the ANN-SVM and ANN-RF models stood out among the other hybrid models as they were positioned farthest from the computed or reference WQI values during the training and validation phases, respectively. The ANN standalone and ANN-SVM models were found closest to the reference point during the training and validation phases, respectively. Taylor diagram considers factors such as standard deviation (SD), correlation (PCC), and root mean square error (RMSE) of the model. It is worth noting that the most effective model is the one that excels in predicting the test dataset, as demonstrated in previous studies [31,66,68,72]. Furthermore, this reaffirms that SVM algorithms enhance the performance of ANN through hybrid models and prove to be superior to all other hybrid and standalone ANN models for predicting WQI in the Bagh River Basin, India.

## 4 Discussion

As detailed above, Sections 3.1, 3.2 describe the WQI prediction results and the key factors that significantly influence the water quality that we have selected for the present study. These factors play a crucial role in shaping the overall water quality in the Bagh River Basin. One important aspect to consider is the computation of the Water Quality Index (WQI), a comprehensive indicator of water quality. Calculating the WQI can be a complex and time-consuming due to the numerous parameters and variables involved. Notably, the values of WQI can vary depending on the specific combination of input parameters used in the calculation. This variability in results is an essential consideration when interpreting WQI values, as highlighted in the work of [73].

To improve the accuracy of WQI assessments, it's often beneficial to include a wide range of input parameters in the analysis, as indicated by research findings by Tiwari et al. [74]. A more comprehensive set of input parameters provides a more holistic view of water quality, leading to a more realistic representation of the WQI. In contrast, it required more lab analysis to compute all the water quality parameters, which is time-consuming and costly. The present study developed and evaluated a new hybrid model (ANN-SVM) to improve the performance of the ANN model. The results of this investigation have demonstrated that Support Vector Machines (SVM) prove to be a highly effective method for addressing a range of environmental issues, as proven in various studies [[75], [76], [77]].

The present study investigated the ANN stand alone and its hybrid five ML models were suitable for predicting WQI (i.e., ANN-RF, ANN-SVM, ANN-RSS, ANN-AR, and ANN-M5P). Based on the Nash-Sutcliffe efficiency (NSE) and root mean squared error (RMSE) in the testing data sets, the order of models' performance for WQI during the testing period was found as ANN-SVM (0.879, 27.974) > ANN (0.842, 31.923) > ANN-M5P (0.782, 37.499) > ANN-RSS (0.742, 40.804) > ANN-AR (0.637, 48.405) > ANN-RF (0.625, 49.224). The results from the machine learning models show that the ANN-SVM model greatly reduces the overall residual errors resulting from the model's accuracy in predicting the future, as shown in Table 4. The residuals of other machine learning models are larger than those of the ANN-SVM and ANN models, which implies that these other machine learning models are ineffective in accurately estimating the field data due to their larger residuals.

The findings of our study align with Nafsin and Li [78] implied the use of a variety of individual machine learning models, including the random forest (RF), artificial neural network (ANN), gradient boosting machine (GBM), support vector machine (SVM), and ensemble-hybrid models such as GBM-SVM, RF-SVM, RF-ANN, ANN-SVM, and RF-GBM for predicting total organic carbon (TOC) and E. coli in the Milwaukee River system. The outcome shows that the ensemble-hybrid model ANN-GBM performed better in forecasting for TOC and E. coli than other models. The effectiveness of six novel hybrid algorithms, including RF-SVM, ANN-SVM, GBM-SVM, RF-ANN, and GBM-ANN, for predicting the BOD of the Buriganga river system in Bangladesh was also examined in a different study. These algorithms included RF-SVM, ANN-SVM, GBM-SVM, RF-ANN, and RF-GBM. One of the study's main findings was the development of a novel hybrid model, the RF-SVM, which has the greatest R^2^ value (0.908) and led to higher prediction success. Another study, Singh et al. [79] highlighted the ANN's potential in predicting WQI. Chou et al. [80] compared four ML algorithms for water quality assessment in Taiwanese reservoirs, finding the ANN model to outperform others. Song et al. [81] showed RF's superior prediction accuracy for pressure ulcer modeling compared to SVM, DT, and ANN. Similarly, Castrillo and García [8] favored the RF model over linear regression for nutrient concentration prediction. Lastly, Nafi et al. [24] found RF more accurate than RT for water quality based on precision, accuracy, and recall metrics. The results from the current investigation also found that the ANN and its hybrid model ANN-SVM have a greater predictive capability for water quality indices in the study area. The new hybrid machine learning model that developed can be particularly useful, especially in developing countries, for efficient and methodical data supervision, water pollution control, prediction of hydrological events, and hydro-chemical parameters forecasting and prevention of hazards. However, hybrid AI models have not always been successful in improving the prediction power of standalone models, and in some cases, they were unable to do so either [23]. The present study has not only identified the key drivers of water quality but has also emphasized the importance of considering a broad spectrum of input parameters when calculating the WQI. Adopting modern soft computing techniques also underscores the potential for more efficient and accurate water quality assessments in the Bagh River Basin and similar regions.

**Fig. 10:**
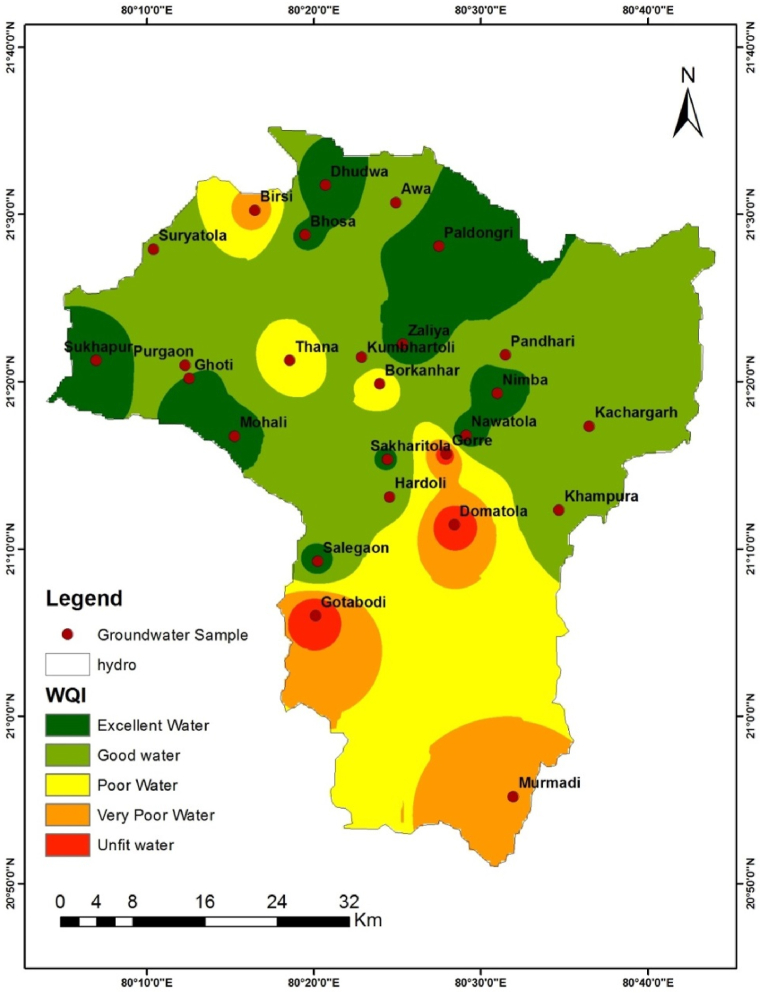
Spatial distribution of WQI in the study river basin.

The suitability of the Bagh River Basin (BRB), a major tributary of the Wainganga River, for irrigation purposes was assessed in this study. We employed the Water Quality Index (WQI) technique to evaluate the quality of irrigation water in the river. The spatial distribution of the WQI map for the Bagh River, generated using GIS, is depicted in Fig. 10. The WQI was categorized into five levels for irrigation purposes: excellent water, good water, poor water, extremely poor water, and unsuitable water. At the Gotobodi and Domatola sampling sites along the Bagh River, a few locations were found to have high WQI levels falling into the "Unsuitable water" category (Fig. 10). It is not advisable to use this water for irrigation. Gotobodi and Domatola recorded the highest WQI values of 376.64 and 369.87, respectively. Generally, as water quality deteriorates, WQI levels increase. The upper reaches of the Bagh River, including areas such as Sukhapur, Ghoti, Mohali, Salegaon, Sakharitola, Gore, Nawatola, Nimba, Zaliya, Paldongri, Bhosa, and Dhudwa, were found to have excellent quality irrigation water. WQI values below 100 indicate that the water is suitable for irrigation in these areas. Good quality irrigation water was observed in the midstream of the Bagh River, particularly in locations like Suryatola, Purgaon, Awa, Kumbhartoli, Pandhari, Kachargarh, Khampura, and Hardoi. However, the water quality was very poor in some areas like Birsi, Thana, Borkanhar, and Murdami villages, as indicated in Fig. 10.

The ML algorithms require large datasets for training and testing, but often water quality data are scarce and expensive to obtain. In addition, water quality is affected by various natural and anthropogenic factors, which can make it challenging to collect and interpret data. Therefore, it is important to ensure that the data used to train ML models are accurate, reliable, and representative of the actual water quality conditions. The ML-based WQI prediction has the potential to provide valuable insights into water quality, particularly in areas where traditional monitoring methods are not feasible or cost-intensive. Moreover, ML models can be used to identify the specific factors that are driving water quality degradation, which can help inform targeted and effective management strategies. Therefore, further research is needed to address the practical and technical challenges associated with ML-based WQI prediction and to develop reliable and interpretable models that can be used for decision-making purposes.

## 5 Conclusions

The present study proposed a new hybrid model (ANN-SVM) using stacked hybridization to improve the performance of Artificial Neural Networks (ANN) in predicting water quality index (WQI) in the Bagh River Basin, India. The approach developed in the present study uses stacking hybridization to combine various machine learning algorithms. The successful integration of the support vector machine (SVM) with ANN and the use of the Relief algorithm to choose the water quality input parameters that have the greatest influence show improved predictive capabilities with high values of Nash-Sutcliffe efficiency (NSE), Pearson correlation coefficient (PCC), and Coefficient of determination (R2), and low values of Mean absolute error (MAE), Root mean square error (RMSE), Relative root square error (RRSE), Relative absolute error (RAE), and Mean squared Error (MSE). The results obtained were further analyzed and compared using graphical representations to facilitate comprehension. It was observed that, with the exception of SVM, none of the other algorithms demonstrated an enhancement in the performance of ANN. During the validation phase, the model performances were ranked as follows: ANN-SVM (NSE = 0.879) > ANN (NSE = 0.842) > ANN-M5P (NSE = 0.782) > ANN-RSS (NSE = 0.742) > ANN-AR (NSE = 0.637) > ANN-RF (NSE = 0.625). These findings offer significant promise for bolstering informed decision-making in water resource management, pollution control, and environmental conservation efforts.

Moreover, the methodology outlined in this study can serve as a valuable framework for refining ANN models across diverse environmental applications, thereby contributing to sustainable development and resource preservation. The present study solely relies on water samples collected within the boundaries of the river basin. Therefore, future research efforts will focus on applying the enhanced AI model across various basins and under diverse climatic conditions to obtain more generalized conclusions.

## Declaration

**Ethics approval:** All authors comply with the guidelines of the journal “*Heliyon”*.

**Consent to participate:** All authors agreed to participate in this study.

**Consent to publication:** All authors agreed to the publication of this manuscript.

## Funding

No funding was received for conducting this study.

## Data availability statement

The data pertaining to this study have not been deposited in a publicly accessible repository, given that all relevant data are thoroughly detailed in the article or appropriately cited in the manuscript.

## CRediT authorship contribution statement

**Nand Lal Kushwaha:** Writing – review & editing, Writing – original draft, Validation, Funding acquisition, Formal analysis, Data curation. **Nanabhau S. Kudnar:** Writing – original draft, Validation, Methodology, Data curation. **Dinesh Kumar Vishwakarma:** Writing – original draft, Software, Investigation, Data curation. **A. Subeesh:** Validation, Resources, Investigation, Conceptualization. **Malkhan Singh Jatav:** Visualization, Software, Methodology, Data curation. **Venkatesh Gaddikeri:** Validation, Project administration, Funding acquisition, Conceptualization. **Ashraf A. Ahmed:** Writing – review & editing, Writing – original draft, Funding acquisition. **Ismail Abdelaty:** Writing – review & editing, Writing – original draft, Visualization, Methodology, Conceptualization.

## Declaration of competing interest

The authors declare that they have no known competing financial interests or personal relationships that could have appeared to influence the work reported in this paper.
